# 6-Gingerol Protects Heart by Suppressing Myocardial Ischemia/Reperfusion Induced Inflammation via the PI3K/Akt-Dependent Mechanism in Rats

**DOI:** 10.1155/2018/6209679

**Published:** 2018-11-05

**Authors:** Tongtong Xu, Guowei Qin, Wei Jiang, Ying Zhao, Yongnan Xu, Xiangwei Lv

**Affiliations:** ^1^Department of Integrated Traditional Chinese and Western Medicine, First Affiliated Hospital of Guilin Medical University, Guilin 541001, Guangxi Zhuang Autonomous Region, China; ^2^Department of Science and Technology, Guilin Medical University, Guilin 541004, Guangxi Zhuang Autonomous Region, China; ^3^Department of Traditional Chinese Medicine, First Affiliated Hospital of Guilin Medical University, Guilin 541001, Guangxi Zhuang Autonomous Region, China; ^4^Department of Respiratory Medicine, Traditional Chinese Medicine Hospital, Xuzhou 221009, Jiangsu, China; ^5^Department of Infectious Diseases, First Affiliated Hospital of Guilin Medical University, Guilin 541001, Guangxi Zhuang Autonomous Region, China

## Abstract

Our previous study has demonstrated that 6-Gingerol (6-G) could alleviate myocardial ischemia/reperfusion injury (MIRI). However, the molecular mechanism underlying the process of myocardial ischemia/reperfusion (I/R) injury alleviation by 6-G remains unelucidated. The objective of the present study is to further investigate the potential mechanism for 6-G to alleviate MIRI in rats. Thirty-two Sprague-Dawley rats were randomly divided into four groups: the Sham group, the I/R group, the 6-G + I/R group, and the LY294002 (LY) + 6-G + I/R group. For the rats in each of the groups, data were collected for cardiogram, cardiac function, area of myocardial infarction, myocardial pathology, myocardial enzyme, marker of inflammatory response, and PI3K/Akt signaling pathway. We found that the pretreatment of 6-G with 6 mg/kg could shrink the ST section of cardiogram, improve the cardiac function, reduce the area of myocardial infarction and the degree of cardiac pathological injury, lower the level of myocardial enzyme, and inhibit the inflammatory response. In addition, our results also indicated that 6-G could upregulate the expression of PI3K and p-Akt and that LY294002, a blocking agent of PI3K/Akt signaling pathway, could nullify the protecting role of 6-G. Our experimental results showed that 6-G could inhibit I/R-induced inflammatory response through the activation of the PI3K/Akt signaling pathway.

## 1. Introduction

Acute myocardial infarction (AMI) is the main cause for the incidence and death of coronary heart disease (CHD) in the world [1]. In-time reperfusion is a key strategy for the treatment of AMI. At present, thrombolytic therapy or percutaneous coronary intervention (PCI) is still the clinically most effective therapy, especially for patients with high ST section [2]. Nevertheless, in the same time of saving lives, reperfusion can also lead to the death of myocardial cell and induce abnormal cardiac function, i.e., myocardial ischemia/reperfusion injury (MIRI) [3, 4].

MIRI is a complex pathophysiological process although its regulatory mechanisms still remain unknown. It has been found that inflammatory response, oxidative induction, and apoptosis all play core roles in the incidence and development of MIRI [5]. Inflammation participates in the pathophysiological process of a variety of cardiovascular diseases, such as myocardial infarction (MI), cardiac hypoxia/reoxygenation (H/R) injury, MIRI, and ischemic heart diseases [6]. Increasing evidences confirmed that inflammation plays a crucial role in MIRI, and it has been proven to be one of the markers for ischemia/reperfusion (I/R) injury. I/R could induce local or systemic massive release of inflammatory cytokines and proinflammatory cytokines, such as TNF-a, IL-6, IL-1*β*, NLRP3, and caspase-1, and further introduce cardiac injury [7, 8]. Likewise, inhibition of I/R-induced inflammatory response can alleviate MIRI [9, 10]. Therefore, inhibition of inflammatory response has become one of the strategies for the clinical treatment of AMI and MIRI.

Phosphoinositide-3 kinase (PI3K) is an important molecule for signal transduction during cell metabolism, while serine/threonine kinase (Akt) is the main target molecule at its downstream, which regulates the survival and death of cells through formation of the PI3K/Akt signaling pathway [11]. In recent years, it has been confirmed that PI3K/Akt signaling pathway plays very important roles in myocardial protection through regulation of inflammatory response, and its activation could inhibit apoptosis and alleviate MIRI [12, 13].

Ginger is an ancient traditional medical and edible plant. It has been used for the treatment of diseases for more than 2000 years in China. Gingerol is a phenolic material in ginger, and 6-gingerol (6-G) is its main ingredient that has marked anti-inflammatory, antioxidative, and antiapoptotic roles [14–16]. Recent studies showed that 6-G could reduce the levels of inflammatory markers, TNF-*α*, IL-6, and IL-1*β*, representing a potential strategy for the treatment of atherosclerosis [17]. Previously, we found that 6-G could alleviate MIRI, together with activation of PI3K/Akt signaling pathway [18].

In this study, we investigated the role of 6-G in myocardial protection by inhibition of inflammatory response and further examined whether 6-G participated in the PI3K/Akt-dependent mechanism.

## 2. Materials and Methods 

### 2.1. Materials and Reagents

6-G (purity, ≥95%) was purchased from Sigma Chemical Co. (MO, USA). Antibodies PI3K, Akt, p-Akt, LY294002 (LY), and *β*-tubulin were supplied by Cell Signaling Technology (MA, USA). TNF-a, IL-6, IL-1*β*, NLRP3, and caspase-1 were provided by Abcam (Cambridge, UK). 2,3,5-triphenyltetrazolium chloride (TTC), BCA protein quantification kit, and the second antibody were purchased from Beyotime (Changsha, China). Cardiac troponin T (cTnT), creatine kinase-MB (CK-MB), tumor necrosis factor-a (TNF-a), interleukin-6 (IL-6), and interleukin-1*β* (IL-1*β*) enzyme-linked immunosorbent assay (ELISA) were acquired from Cusabio Biotech Co. (MD, USA).

### 2.2. Animal and Ethics

Sprague-Dawley (SD) male rats, weighed 200-250g, were supplied by the experimental animal center of Guangxi Medical University (Execute No.: SCXK [Gui] 2014-0003.) All protocols and agreements dealing with the experiments were approved by the Ethic Commission of Experimental Animals, Guangxi Medical University. The experimental animals were raised under standard experimental conditions, 20°C-25°C, 50%-60% RH, and 12L/12D photoperiod. The experimental rats were fed with clean drinking water and food* ad libitum*.

### 2.3. Animal Model and Experimental Design

Thirty-two SD rats were randomly divided into four experimental groups: (1) Sham group: this group of rats accepted thoracotomy, but their left anterior descending (LAD) coronary arteries were not ligated; (2) I/R group: these rats accepted thoracotomy, and their LADs were ligated for 30 min and then reperfused for two hours; (3) 6-G + I/R group: 30 min before LAD ligation, rats in this group were injected with 6-G (6mg/kg) via tail vein and then reperfused for 2 hours; (4) LY + 6-G + I/R group: 30 min before LAD ligation, rats in this group were injected with LY294002 (0.3mg/kg) and 6-G and then reperfused for 2 hours. The experimental rats were paralyzed with pentobarbital sodium (40 mg/kg), and their respiration was maintained with respirator at 80-90 cycles/min. The inspiration/expiration was 1:2 and the tidal volume was 8-10 mL/kg. Thoracotomy was performed at the fourth rib of left sternum to expose heart. LAD was ligated for 30 min with 6-0 suture line and then reperfused for 2 hours. Limb leads of electrocardiography were inserted into animal's limbs to record the changes of electrocardiogram. The observations of elevated ST section in the electrocardiogram as well as of gray and tumid myocardium at the ligated blood supply area serve as the criteria for successful model construction.

### 2.4. Ultrasonic Electrocardiogram Study

After successful model construction, changes in rat cardiac function in each group were evaluated using an invasive hemodynamics method. Left ventricular ejection fraction (EF), left ventricular fractional shortening (FS), left ventricular end systolic diameter (LVESD), left ventricular end diastolic diameter (LVEDd), and maximum rise/down velocity of left intraventricular pressure (±dp/dtmax) were recorded with the MS400 biological signal analysis system (Longfeida Technology Co., Ltd., Shandong, China). The mean values of the three cardiac cycles were used for statistical analysis.

### 2.5. Determination of the Area of Myocardial Infarction by 2,3,5-Triphenyltetrazolium Chloride

The area of MI was quantified by 1% of TTC. After reperfusion rat heart was removed and blood washed with PBS solution. The heart was kept in freezer at −20°C for 20 min and then cut into 5 mm thick specimen. The resulting specimens were soaked in 1% of TTC solution and then incubated at 37°C for 30 min. White and gray white represent area of infarction, and red represents area of noninfarction. The area of infarction was determined by the Image-Pro Plus 6.0 software.

### 2.6. Cardiac Histopathology by Hematoxylin and Eosin

After reperfusion, rat hearts were removed quickly and the blood was washed away with PBS solution. The heart was soaked in 4% of paraformaldehyde, imbedded in paraffin, cut into 2 mm thick slices, and then subjected to Hematoxylin and Eosin (H&E) staining examination. Myocardial injury was observed under an optic microscope. Each sample was observed at ×400 magnification. The cardiac myocardial injury was scored according to the following standard: Score 0: no myocardial structure injury; Score 1: slight myocardial mesenchymal edema, gap increase, and local necrosis; Score 2: extensive myocardial swell and mesenchymal edema, and medial local necrosis; Score 3: severe small vessel damage and myocardial necrosis, massive inflammatory cellular infiltration, and formation of contraction bands; Score 4: severe diffusive myocardial necrosis and hemorrhage, accompanied by massive small vessel damage and formation of contraction bands.

### 2.7. Determination of Serums Myocardial Enzyme by ELISA

Two hours after reperfusion, arterial blood was collected at apex cordis with heparin anticoagulation tube and then centrifuged by 8000g at 4°C for 10 min. Supernatant was collected to determine the levels of cTnT and CK-MB with ELISA reagent kit.

### 2.8. Serums Inflammation Marker and ELISA Test

The content of TNF-a, IL-6, and IL-1*β* (markers of inflammatory response) in supernatant was quantified with ELISA reagent kit.

### 2.9. Analysis of Cardiac Muscle Protein by Western Blot

150 mg of myocardial tissue was collected from each group and broken with ultrasonic grinder. RIPA buffer containing phenylmethylsulfonyl fluoride (PMSF) was added to the homogenate and then kept on ice for 30 min to sufficiently lyse the tissue. Subsequently, the lysate was inserted into a 2-ml centrifugal tube and then centrifuged by 12000g at 4°C for 15 min. Protein content was quantified with BCA kit. The protein was subjected to SDS-PAGE and then transferred to the PVDF membrane. The PVDF membrane was incubated by PI3K, Akt, p-Akt, TNF-a, IL-6, IL-1*β*, NLRP3, caspase-1, and *β*-tubulin for 6 h and then by the second antibody for 4 h. The data was analyzed with the chemiluminescence system (Amersham Pharmacia).

### 2.10. Data Analysis

SPSS statistical package (version 21.0; IBM, NY, USA) was used to analyze the data using Student's t-test or one-way ANOVA. The data were expressed as mean value ± standard deviation, and* P *< 0.05 represent statistical significance. 

## 3. Results

### 3.1. Changes of Electrocardiogram in I/R Model and Effects of 6-G Pretreatment on Electrocardiogram

**Figure 1:**
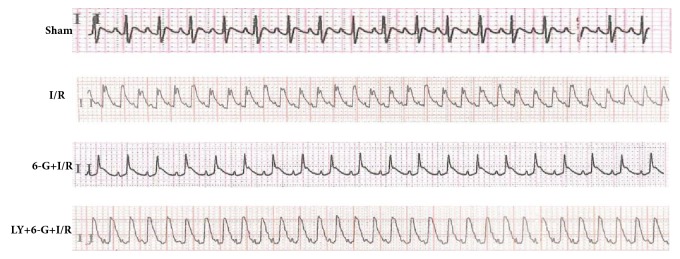
ECG changes in successful I/R rat model and effects of 6-G treatment on ECG (n=8 for each group).

As shown in Figure 1, no significant change in EGG was observed for the Sham group. The ST section for the I/R group was significantly increased, suggesting successful construction of cardiac I/R model. It can also be seen that 6-G pretreatment significantly reduced the ST section while LY294002 could offset the curative effect of 6-G.

### 3.2. 6-G Pretreatment Improved the Cardiac Function of I/R Rats

**Figure 2:**
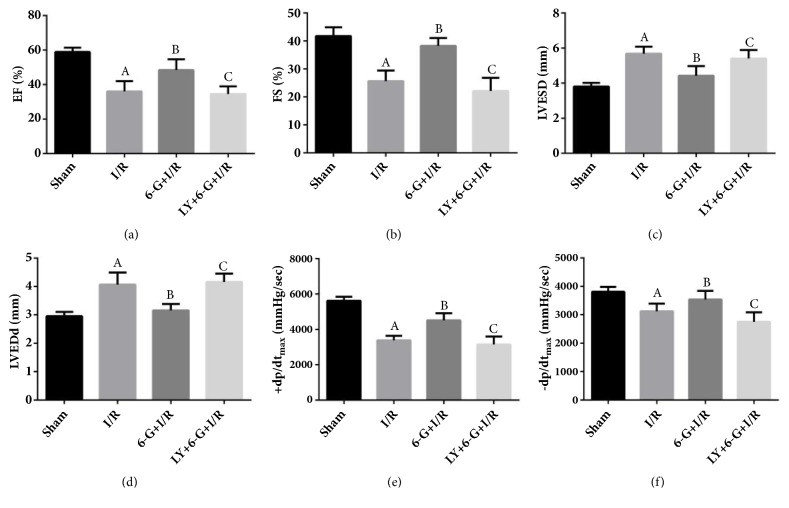
6-G treatment could improve I/R-induced disorder of cardiac function, but this effect could be offset by LY294002 (n=8 for each group). Note that ^*A*^*P *< 0.05 against the Sham group; ^*B*^*P *< 0.05 against the I/R group; ^*C*^*P *< 0.05 against the 6-G + I/R group.

As illustrated in the data plotted in Figure 2, the EF, FS,* +dp/dt*_max_, and* -dp/dt*_max_ in I/R group were significantly increased comparing to that in the Sham group. 6-G pretreatment significantly increased EF, FS,* +dp/dt*_max_, and* -dp/dt*_max_ (Figures 2(a), 2(b), 2(e), and 2(f)), but reduced LVESD and LVEDd (Figures 2(c) and 2(d)). Meanwhile, the curative effect of 6-G could be reversed by LY294002.

### 3.3. Reduction of I/R-Induced Myocardial Infarction Area by 6-G Pretreatment

**Figure 3:**
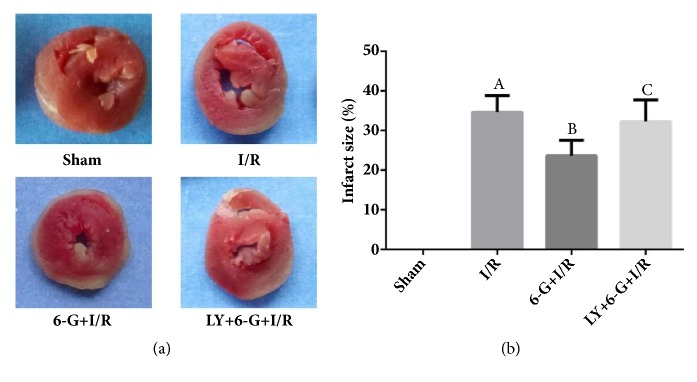
6-G pretreatment can shrink the area of I/R-induced MI, but this biological function can be reversed by LY294002 (n=8 for each group). Note that ^*A*^*P *< 0.05 against the Sham group; ^*B*^*P *< 0.05 against the I/R group; ^*C*^*P *< 0.05 against the 6-G + I/R group.

Our study also observed that the area of MI in the I/R group was significantly increased relative to that in the Sham group. As shown in Figures 3(a) and 3(b), 6-G treatment could shrink the area of myocardial infarction. However, this pharmacological activity of 6-G can be reversed by LY294002.

### 3.4. Alleviation of IR-Induced Myocardial Injury by 6-G Pretreatment

**Figure 4:**
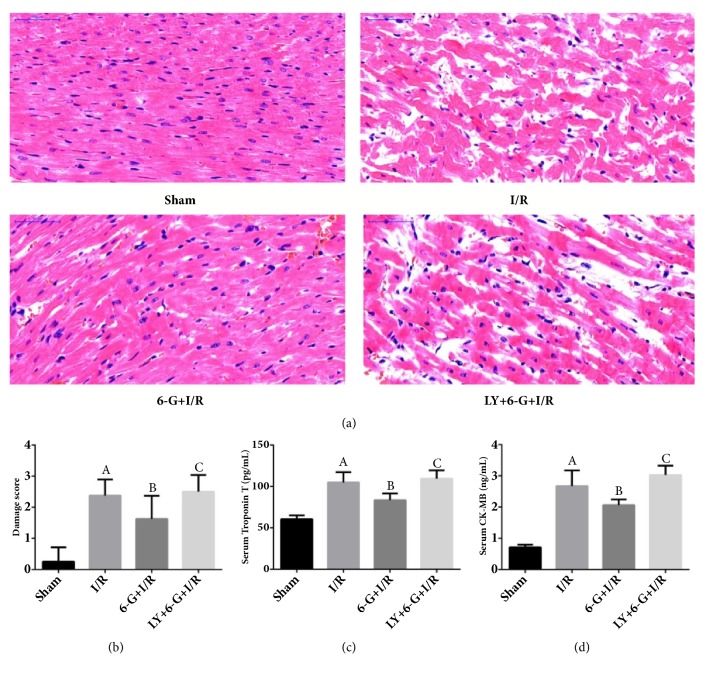
6-G treatment could alleviate myocardial injury and reduce the level of markers of myocardial injury, but LY294002 could reverse the protecting role of 6-G in myocardial (n=8 for each group). Note that ^*A*^*P *< 0.05 against the Sham group; ^*B*^*P *< 0.05 against the I/R group; ^*C*^*P *< 0.05 against the 6-G + I/R group.

Results of H&E staining test showed that, comparing with Sham group, the myocardial injury in the I/R group was significant while the degree of myocardial injury was positively correlated with the changes in cTnT and CK-MB (markers of myocardial injury) as shown in Figures 4(a), 4(b), 4(c), and 4(d). 6-G pretreatment could alleviate I/R-induced myocardial injury and decrease the level of markers of myocardial injury, suggesting that 6-G could alleviate the effect of myocardial injury. However, LY294002 could reverse the effect of 6-G by blocking the PI3K/Akt signaling pathway.

### 3.5. 6-G Pretreatment Could Alleviate I/R-Induced Inflammatory Response

**Figure 5:**
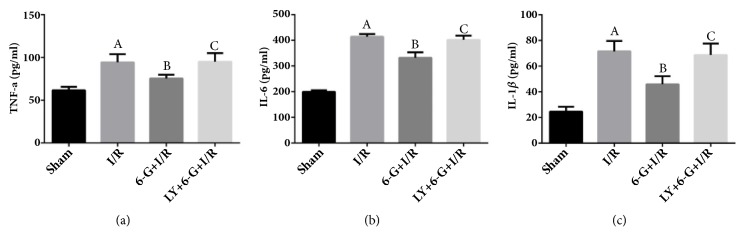
6-G treatment could alleviate I/R-induced inflammatory response, but LY294002 could reverse this effect (n=8 for each group). Note that ^*A*^*P *< 0.05 against the Sham group; ^*B*^*P *< 0.05 against the I/R group; ^*C*^*P *< 0.05 against the 6-G + I/R group.

**Figure 6:**
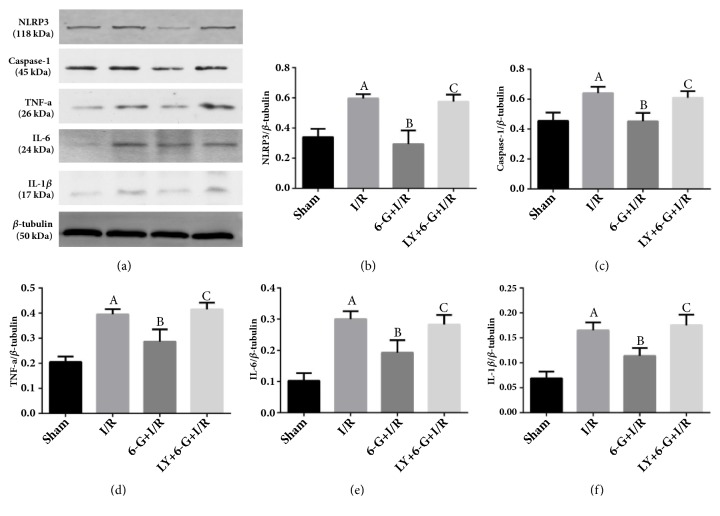
6-G treatment could inhibit myocardial inflammatory response, but LY294002 could reverse the anti-inflammatory effect of 6-G (n=8 for each group). Note that ^*A*^*P *< 0.05 against the Sham group; ^*B*^*P *< 0.05 against the I/R group; ^*C*^*P *< 0.05 against the 6-G + I/R group.

① Results of ELISA assay showed that the levels of serums inflammatory markers, TNF-a, IL-6, and IL-1*β*, in the I/R group were significantly increased compared to those in the Sham group. As shown in Figures 5(a), 5(b), and 5(c), 6-G treatment could reduce the levels of TNF-a, IL-6, and IL-1*β*. However, we also found that LY294002 could reverse this beneficial effect of 6-G.

② Results of Western blot test showed that the expression levels of myocardial inflammation markers, TNF-a, IL-6, IL-1*β*, NLRP3, and caspase-1, in the I/R group were significantly increased compared to those in the Sham group. As shown in Figures 6(a), 6(b), 6(c), 6(d), 6(e), and 6(f), 6-G pretreatment could reduce the expression levels of I/R-induced myocardial inflammation markers. However, LY294002 could offset 6-G-inhibited myocardial inflammation response by blocking the PI3K/Akt signaling pathway.

### 3.6. Effect of PI3K/Akt Signaling Pathway on the Inhibition of Myocardial Inflammation Response by 6-G Pretreatment

**Figure 7:**
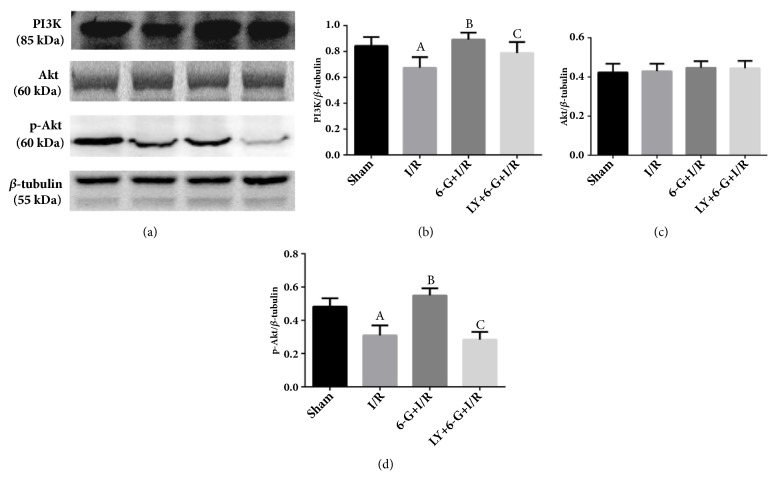
6-G pretreatment could activate the PI3K/Akt signaling pathway, but LY294002 could block this effect of 6-G (n=8 for each group). Note that ^*A*^*P *< 0.05 against the Sham group; ^*B*^*P *< 0.05 against the I/R group; ^*C*^*P *< 0.05 against the 6-G + I/R group.

Results of Western blot assay showed that the expression levels of PI3K and p-Akt for the I/R group were significantly reduced as compared to the Sham group. As shown in Figures 7(a), 7(b), and 7(d), 6-G treatment could increase the expression of PI3K and p-Akt. However, LY294002, a blocking agent of the PI3K/Akt signaling pathway, could offset the activation role of 6-G via the PI3K/Akt signaling pathway. These results showed that 6-G could inhibit myocardial inflammation response and alleviate MIRI by the PI3K/Akt-dependent mechanism.

## 4. Discussion

In the present study, we found that 6-G could reduce the ST section of I/R-induced electrocardiogram, improve cardiac function, shrink the area of MI, alleviate cardiac pathological injury, decrease the levels of markers of cardiac injury (cTnT and CK-MB), attenuate the level of serums inflammatory markers, TNF-a, IL-6, and IL-1*β*, and inhibit the expression of cardiac inflammatory markers, TNF-a, IL-6, IL-1*β*, NLRP3, and caspase-1. Furthermore, we observed that 6-G could inhibit cardiac inflammatory response and alleviate MIRI through the PI3K/Akt-dependent mechanism. To our knowledge, it is the first time that such result is reported by direct in vivo experiment.

Previous study showed that 6-G could inhibit the activation of angiotensin II type 1 receptor (AT_1_), functioning to regulate blood pressure and to enhance myocardial contractility [19]. In addition, 6-G could increase the activity of cardiac sarcoplasmic reticulum Ca^2+^-ATPase and enhance myocardial contractility [20]. In the present study, we also found that 6-G could reduce the ST section of I/R-induced electrocardiogram, while cardiac function has been significantly improved.

I/R-induced cardiac injury is a complicated pathophysiological process, with the involvement of multiple layers of the body, organs, cells, and molecules, as well as multiple pathways of genes, proteins, and signaling pathways [21]. It has been confirmed that excessive inflammatory response and oxidative induction during reperfusion are the key factors that lead to cardiac cell death [22, 23]. More researches have suggested that nucleotide-binding oligomerization domain-like receptor family pyrin domain-containing 3 (NLRP3) inflammasome has participated in the pathophysiological process of MIRI, and inflammasome is a key factor that regulates IL-1*β* and activate caspase-1; inhibition of NLRP3 could shrink the area of myocardial infarction and the reconstruction of left ventricle following infarction [24, 25]. I/R-mediated oxidative induction further leads to the release of proinflammatory cytokines, IL-6 and TNF-a. These proinflammatory cytokines not only injure cardiac tissue locally, but also are released into the circulatory system leading to systemic injury [26, 27]. Therefore, a reduction of the levels of TNF-a and IL-6 could alleviate MIRI [7, 28, 29].

In our present study, we also revealed that 6-G could reduce the level of serums inflammatory markers, TNF-a, IL-6, and IL-1*β*, and inhibit the expression of myocardial inflammatory markers TNF-a, IL-6, IL-1*β*, NLRP3, and caspase-1. More importantly, 6-G could reduce the levels of I/R-induced markers of myocardial injury, cTnT and CK-MB, alleviate myocardial pathological injury, and shrink the area of myocardial infarction.

PI3K/Akt is a famous signaling pathway that functions to protect myocardial muscle. It regulates the survival and death of myocardial muscle during the I/R period [30–32]. Increasingly more results have suggested that the activation of the PI3K/Akt signaling pathway could inhibit I/R-induced inflammatory response and alleviate oxidative induction and apoptosis [23, 33].

It was also reported that B-type natriuretic peptide (BNP) could inhibit inflammatory response and alleviate MIRI through activation of the PI3K/Akt signaling pathway MIRI [13]. Further, (S)-1-(a-naphthylmethyl)-6,7-dihydroxy-1,2,3,4-tetrahydro isoquinoline (CKD712) could also reduce myocardial inflammation and level of apoptosis and alleviate MIRI through the same pathway [34]. In addition, Pitavastatin could inhibit inflammatory response and alleviate MIRI through the PI3K/Akt-dependent mechanism [35].

In order to confirm whether 6-G could protect myocardial muscle through the PI3K/Akt-dependent mechanism, we used LY294002 (a blocking agent of the PI3K/Akt signaling pathway) to further set forth its molecular mechanism. Our results showed that 6-G could both inhibit the inflammatory response and increase significantly the expression levels of PI3K and p-Akt. However, this beneficial effect could be reversed by LY294002. Our data showed that 6-G inhibits inflammatory response and alleviates MIRI in a PI3K/Akt-dependent mechanism.

This study also has certain limitations. Although we used LY294002 to block the PI3K/Akt signaling pathway, however, we do not know whether LY294002 has an effect on the myocardium. In the next stage, we will set the LY294002 group, the LY294002 + I/R group, and the LY294002 + 6-G group for observation.

## 5. Conclusion

Our results demonstrated that 6-G pretreatment could play the anti-inflammatory role and alleviate I/R-induced myocardial injury through the PI3K/Akt-dependent mechanism. Therefore, 6-G is potentially a candidate to be used in attempts for the treatment of AMI and MIRI.

## Data Availability

The data of this study are supported by the Natural Science Foundation of China and therefore cannot be used free of charge. Access to these data requires permission from Tongtong Xu, which the author will consider upon request.
