# Corrigendum to “6-Gingerol Protects Heart by Suppressing Myocardial Ischemia/Reperfusion Induced Inflammation via the PI3K/Akt-Dependent Mechanism in Rats”

**DOI:** 10.1155/2019/7659701

**Published:** 2019-06-18

**Authors:** Tongtong Xu, Guowei Qin, Wei Jiang, Ying Zhao, Yongnan Xu, Xiangwei Lv

**Affiliations:** ^1^Department of Integrated Traditional Chinese and Western Medicine, First Affiliated Hospital of Guilin Medical University, Guilin 541001, Guangxi Zhuang Autonomous Region, China; ^2^Department of Science and Technology, Guilin Medical University, Guilin 541004, Guangxi Zhuang Autonomous Region, China; ^3^Department of Traditional Chinese Medicine, First Affiliated Hospital of Guilin Medical University, Guilin 541001, Guangxi Zhuang Autonomous Region, China; ^4^Department of Respiratory Medicine, Traditional Chinese Medicine Hospital, Xuzhou 221009, Jiangsu, China; ^5^Department of Infectious Diseases, First Affiliated Hospital of Guilin Medical University, Guilin 541001, Guangxi Zhuang Autonomous Region, China

In the article titled “6-Gingerol Protects Heart by Suppressing Myocardial Ischemia/Reperfusion Induced Inflammation via the PI3K/Akt-Dependent Mechanism in Rats” [1], there was an error in the HE detection image of LY+6-G+I/R group in Figure 4(a).  Figure 4 is shown below.

## Figures and Tables

**Figure 4:**
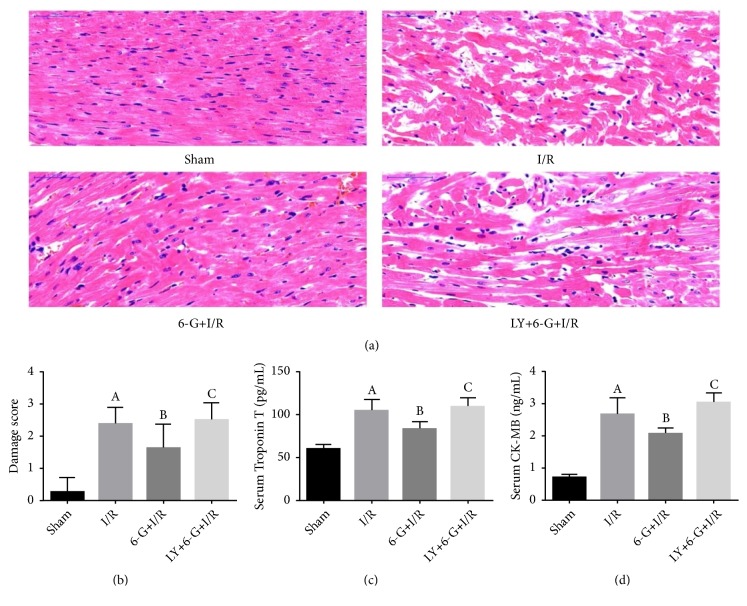
6-G treatment could alleviate myocardial injury and reduce the level of markers of myocardial injury, but LY294002 could reverse the protecting role of 6-G in myocardium (n=8 for each group). Note that ^*A*^*P *< 0.05 against the Sham group; ^*B*^*P *< 0.05 against the I/R group; and ^*C*^*P *< 0.05 against the 6-G + I/R group.
